# Anti-TGF-β1 aptamer enhances therapeutic effect of tyrosine kinase inhibitor, gefitinib, on non-small cell lung cancer in xenograft model

**DOI:** 10.1016/j.omtn.2022.06.001

**Published:** 2022-06-29

**Authors:** Masaki Takahashi, Yoshifumi Hashimoto, Yoshikazu Nakamura

**Affiliations:** 1 Project Division of RNA Medical Science, The Institute of Medical Science, The University of Tokyo, Minato-ku, Tokyo 108-8639, Japan; 2 RIBOMIC Inc., Minato-ku, Tokyo 108-0071, Japan

**Keywords:** oligonucleotides, therapies and applications, aptamer, TGF-β1, isoform specific, gefitinib, non-small cell lung cancer

## Abstract

Transforming growth factor β (TGF-β) is a multifunctional cytokine that plays crucial pathophysiological roles in various diseases, such as cancer and fibrosis. However, the disease modulation by targeting TGF-β1 isoform remains to be established, regardless of several studies employed with limited antibodies. Here, we developed an RNA aptamer to human active TGF-β1, named APT-β1, and characterized its properties *in vitro* and *in vivo*. APT-β1 bound to human and mouse active TGF-β1 proteins with high affinity and specificity and strongly inhibited TGF-β1-induced downstream signaling and cell morphology with 50% inhibition concentration (IC50) values at picomolar concentrations. In a xenograft mouse model of non-small cell lung cancer, APT-β1 alone showed no appreciable effect on tumor growth, while it greatly enhanced the anti-tumor effect of gefitinib, an approved tyrosine kinase inhibitor. These findings strongly suggest that the anti-TGF-β1 medication may be a promising cancer therapy to suppress repopulation of lung cancer in combination with certain anti-cancer drugs, such as gefitinib.

## Graphical abstract

**Figure:**
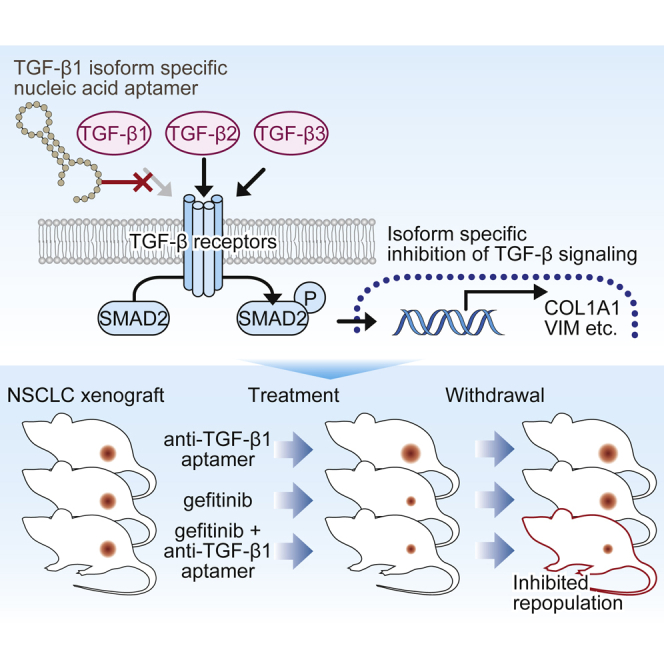

## Teaser

An RNA aptamer specific to active transforming growth factor β1 (TGF-β1) isoform was developed and evaluated for its effects in a lung cancer xenograft model. The aptamer indicated that inhibition of active TGF-β1 might suppress repopulation of lung cancer tumor in combination treatment with certain anti-cancer drugs, such as gefitinib.

## Introduction

Transforming growth factor β (TGF-β) is a pleiotropic cytokine involved in diverse cellular events, including cell proliferation, differentiation, immune response, and apoptosis.^1^^,^^2^ In mammals, there are three isoforms of TGF-β—TGF-β1, -β2, and -β3—encoded at distinct genomic loci,^3^ and TGF-β1 is the most abundant isoform in various tissues.^4^ Although the isoforms largely overlap in their amino acid sequences and functions, *in vivo* studies using isoform-specific knockout mice showed distinct phenotypes,^4^^,^^5^ indicating differential expression profiles and mechanisms of action of the isoforms.^6^, ^7^, ^8^, ^9^ Thus, when considering TGF-βs as therapeutic targets, their roles in physiological events should be considered carefully.

Over the past decades, TGF-β has gained attention as a therapeutic target in various diseases, such as tissue fibrosis and tumorigenesis. In relation to cancer, TGF-β exhibits both anti-tumoral and oncogenic properties; however, many agents blocking the TGF-β signaling pathway have demonstrated promising anti-tumor activity in preclinical studies.^10^^,^^11^ Therapeutic agents targeting distinct components of the TGF-β signaling pathway have been developed and evaluated in clinical trials.^10^^,^^12^ However, single-drug therapy that blocks the TGF-β signaling pathway has not yet shown clear therapeutic evidence in clinical trials.^13^ Some of the trials were even terminated due to unfavorable effects, including dose-limiting toxicities.^14^, ^15^, ^16^ Since TGF-β2 and -β3 serve important functions in the cardiovascular system, such adverse events in the clinical trials might be due to non-selective inhibition of all TGF-β isoforms.^14^^,^^17^, ^18^, ^19^, ^20^, ^21^, ^22^ Hence, considering the expression levels and functions of each isoform, several inhibitors specific to TGF-β1, such as antibodies and antisense oligonucleotides, have been developed and evaluated in preclinical and clinical studies.^15^^,^^23^ The antisense oligonucleotide strategy is a promising approach in terms of specificity but needs further refinement to overcome the major hurdle of efficient drug delivery. Regarding antibody, there are several TGF-β1-specific antibodies (LY2382770, metelimumab, ABBV-151, and SRK-181), but the antibodies have yet to be fully investigated in clinical trials.^15^^,^^24^, ^25^, ^26^, ^27^ Thus, the effects of TGF-β1-specific inhibition on various diseases remain to be elucidated. A novel agent specifically inhibiting TGF-β1 needs to be developed and examined for therapeutic effect of TGF-β1-specific inhibition toward TGF-β-related diseases, including cancers.

In this study, we developed an anti-TGF-β1 RNA aptamer, APT-β1, and examined its effect on a xenograft cancer model using non-small cell lung cancer (NSCLC) cells, in which TGF-β has a significant impact on progression in preclinical and/or clinical studies.^28^ Nucleic acid aptamers specifically bind to target of interest by conforming to a unique tertiary structure and are generated by a process known as the systematic evolution of ligands by exponential enrichment (SELEX).^29^^,^^30^ These molecules are expected to be a new medical modality possessing favorable pharmacological characteristics compared with antibodies because of chemical synthesis, limited antigenicity, and better tissue penetration due to the medium size between antibodies and chemicals.^31^

## Results

### Selection and properties of APT-β1 aptamer

**Figure 1:**
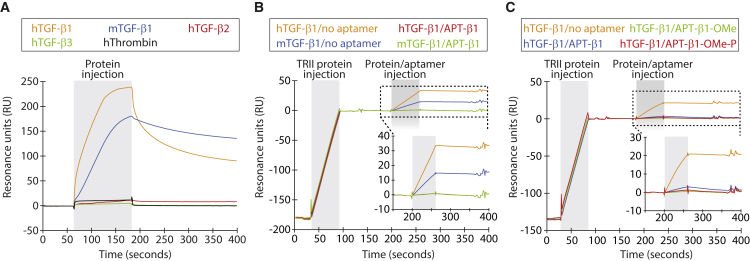
Binding analysis of anti-TGF-β1 aptamers by SPR analysis (A) Affinity of APT-β1 to human three TGF-β isoforms and mouse TGF-β1. The APT-β1 labeled with biotin at 3′ end was immobilized to a streptavidin (SA) sensor chip approximately 60–140 RUs. The indicated proteins (500 nM; human TGF-β isoforms [hTGF-β1–3], mouse TGF-β1 [mTGF-β1], and human thrombin as a negative control) were injected at indicated time periods. (B) The ability of APT-β1 to inhibit the interaction of TGF-β1 with TGF-β receptor type II (TRII) is shown. Human TRII fused with Fc protein (TRII/Fc, 30 nM) was immobilized to a CM5 sensor chip mediating a protein A. The hTGF-β1 (4 nM) or mTGF-β1 (4 nM) was injected in the presence or absence of APT-β1 (8 nM). SPR sensorgrams of TGF-β1 bound to TRII were shown as an enlarged insert. (C) The ability of APT-β1 and its modified sequences to inhibit the interaction of TGF-β1 with the receptor is shown. As in (B), TRII/Fc protein was immobilized and hTGF-β1 (4 nM) was injected in the presence or absence of APT-β1 (4 nM), APT-β1-OMe (4 nM), and APT-β1-OMe-P (4 nM). The sensorgrams showing bindings of TGF-β1 to TRII were shown as an enlarged insert.

APT-β1 was selected against TGF-β1 by SELEX and is composed of 33 nt. Surface plasmon resonance (SPR) analysis showed that APT-β1 binds to human TGF-β1, but not to TGF-β2 and -β3 isoforms, and latent TGF-β1 that is stored forms in the extracellular matrix (Figures 1A and S1). APT-β1 binds to mouse TGF-β1 and inhibited ligand binding to human TGF receptors (Figure 1B). APT-β1 was manipulated by ribose 2′-*O*-methyl (2′-OMe) and 2′-fluoropyrimidine modifications at 18 and 5 positions, respectively, to resist ribonucleases, giving rise to APT-β1-OMe.^32^ APT-β1-OMe was further conjugated with polyethylene glycol (PEG) and inverted dT at 5′-and-3′ termini, respectively, to improve the pharmacokinetic properties. The resulting structure is referred to as APT-β1-OMe-P and able to inhibit the ligand-receptor interaction (Figure 1C). Notably, the binding of TGF-β1 at 4.0 nM concentration to the receptor was completely blocked by APT-β1-OMe with and without PEG at 4.0 nM, indicating the high neutralizing potency of APT-β1-OMe. The dissociation constant (K_D_) value of APT-β1-OMe-P was estimated to be in the nanomolar range, as calculated by the bivalent and univalent binding models, respectively (Figure S2). These results showed the high affinity and specificity of APT-β1-OMe-P toward TGF-β1.

### Inhibition of SMAD2 signaling pathway

**Figure 2:**
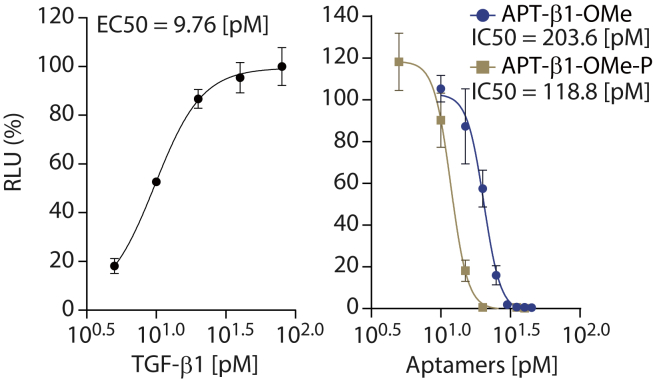
The ability of APT-β1-OMe and APT-β1-OMe-P to inhibit TGF-β1-induced expression of SMAD2-responsive luciferase reporter In the left panel, HEK293 cells transfected with the SMAD2-responsive luciferase reporter plasmid were treated with various amounts of TGF-β1. In the right panel, TGF-β1 (80 pM) was added to HEK293 cells, which were transfected with the reporter plasmid, in the presence or absence of various amounts of APT-β1-OMe-P and APT-β1-OMe. The values were expressed as relative luminescent units (RLUs) (shown in %) to the 100 pM (left panel) or 80 pM TGF-β1 level (right panel) without aptamer after subtraction of basal LUs in control cells without treatment. Data represent the mean ± SD (n = 3).

TGF-β1 induces phosphorylation of SMAD2 via TGF-β receptors. Phosphorylated SMAD2 (p-SMAD2) interacts with SMAD-binding element (SBE) in corresponding promoter sequences on genomic DNA and regulates the expression of various target genes, affecting a wide variety of physiological phenomena.^33^ Thus, we evaluated the effect of APT-β1-OMe with and without PEG by conventional promoter assay using a luciferase reporter in HEK293 cells.^34^ In this analysis, TGF-β1 treatment increased luminescent reporter expression with an 50% effective concentration (EC50) of 9.76 pM (Figure 2, left panel). The reporter expression induced by 80 pM TGF-β1 was blocked by aptamers APT-β1-OMe and APT-β1-OMe-P at 50% inhibition concentration (IC50) values of 203.6 pM and 118.8 pM, respectively (Figure 2, right panel). Further, western blot analysis showed that 300 pM APT-β1-Ome-P was sufficient to abolish phosphorylation of endogenous SMAD2 in HEK293 cells activated by 80 pM TGF-β1 (Figure S3, upper panel). However, anti-pan-TGF-β antibody, which was used as a positive control possessing a neutralizing activity, could not fully suppress phosphorylation of SMAD2 even at 2.6 nM concentration.^35^^,^^36^ One of the APT-β1 derivatives, APT-β1-F, which lost the inhibitory activity against TGF-β1 in the reporter assay (Figure S4), failed to block SMAD2 phosphorylation. Besides, APT-β1-Ome or APT-β1-Ome-P did not affect phosphorylation of endogenous SMAD2 by TGF-β2 and -β3, confirming the specificity of these aptamers to TGF-β1 (Figures S3, lower panel, and S5).

### Effects of APT-β1 on TGF-β1-induced gene expression

**Figure 3:**
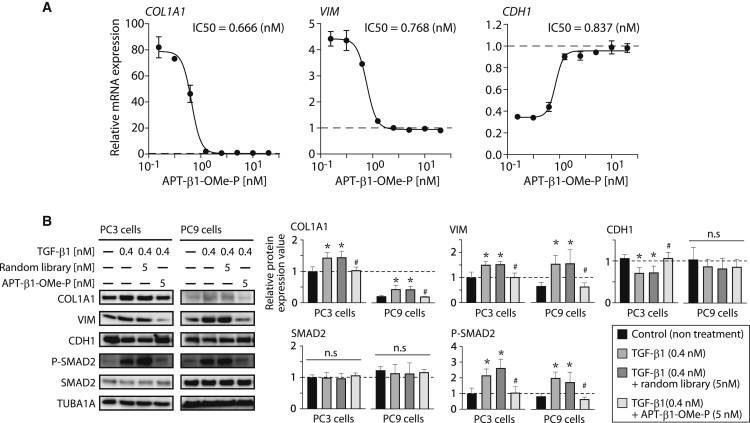
Inhibitory effect of APT-β1-OMe-P on the expression of TGF-β1-induced EMT-related genes in A549 cells and NSCLCs (A) Altered mRNA expression levels of TGF-β1-induced EMT relevant genes by APT-β1-OMe-P. A549 cells were treated with or without TGF-β1 (0.4 nM) in the presence or absence of various amounts of APT-β1-OMe-P for 24 h, and the mRNA expression levels of TGF-β responsive genes (*COL1A1*, *VIM*, and *CDH1*) were examined by real-time PCR. The expression levels of those genes were normalized by the expression levels of *GAPDH*, and then the normalized values of each gene in the various treated cells were expressed as relative expression levels to those in the control cells without any treatments as 1, which is indicated by a dashed line. Data represent the mean ± SD (n = 3). The IC50 values of APT-β1-OMe-P in the expression levels of each TGF-β1-induced EMT-related gene were shown in the graph. (B) Altered protein expression levels of TGF-β1-induced EMT-related genes by APT-β1-OMe-P in NSCLCs are shown. NSCLCs, PC3, and PC9 cells were treated with or without TGF-β1 (0.4 nM) in the presence or absence of APT-β1-OMe-P (5 nM) for 24 h, and the protein expression levels of indicated genes were examined by western blot analysis. Random library (5 nM) was used as a control. The expression levels of those genes were normalized by the expression levels of TUBA1A1, and then the normalized values of each gene in the various treated cells were expressed as relative expression levels to those of control group in PC3 cells without any treatments as 1, which is indicated by a dashed line. Statistical differences among treatment groups were examined by one-way ANOVA and then by Tukey-Kramer test. Data represent the mean ± SD (n = 3). ∗p < 0.05 versus controls in each cell type, #p < 0.05 versus treatment with random library and TGF-β1 in each cell type, n.s, no significant difference between indicated groups.

Reversibility of TGF-β1-induced expression of TGF-β1-responsive genes, *collagen type I alpha 1 chain* (*COL1A1*), *vimentin* (*VIM*), and *E-cadherin* (*CDH1*),^37^ were examined upon addition of APT-β1-OMe-P/APT-β1-OMe in human lung adenocarcinoma A549 cells that were pre-treated with 0.4 nM TGF-β1 (Figure 3A). *COL1A1* and *VIM* upregulated by TGF-β1 were suppressed by APT-β1-OMe-P with IC50s of 0.666 nM and 0.768 nM, respectively, while downregulated *CDH1* was reversed to a basal level with IC50 of 0.837 nM. The same reversal was observed with APT-β1-OMe with IC50 of picomolar range (Figure S6A). The similar trends were observed with anti-pan-TGF-β antibody in a less inhibitory manner with IC50 of mM range (Figure S6B).^35^^,^^36^ Furthermore, the neutralizing effect of APT-β1-OMe-P on TGF-β1-induced changes in expression levels of those genes were also observed at protein level (Figure S7), although the TGF-β1-induced changes in expression of COL1A1, VIM, and CDH1 were apparently less obvious compared with those at mRNA level. In addition, two other TGF-β1-responsive proteins, SNAIL and N-cadherin (CDH2),^37^ were upregulated by TGF-β1 treatment and reversed upon addition of APT-β1-OMe-P (Figure S8). Thus, APT-β1-OMe-P is able to reverse TGF-β1-induced expression at both mRNA and protein levels in A549 cells. Based on these results using major lung cancer cells, we further investigated reversibility of TGF-β1-induced expression of TGF-β1-responsive genes in protein levels in NSCLCs PC3 and PC9 cells by APT-β1-OMe-P (Figure 3B). Although CDH1 in PC9 cells did not show clear reversibility due to its low sensitivity to TGF-β1, the other genes in both cell lines significantly inhibited TGF-β1-induced protein expression changes by APT-β1-OMe-P.

TGF-β1 treatment in A549 cells reportedly induces epithelial-mesenchymal transformation (EMT) accompanied by cellular morphological changes.^38^^,^^39^ We examined alterations in the shape of A549 cells treated with TGF-β1 in the absence or presence of APT-β1-OMe-P (Figure S9A). Consequently, TGF-β1 treatment at 0.4 nM for 2 days induced spindle-shaped alterations of A549 cells as reported previously,^38^^,^^39^ and these changes were prevented by APT-β1-OMe-P at 5 nM. Unlike A549 cells, PC3 and PC9 cells not only showed no obvious changes in cell shape upon TGF-β1 treatment but also showed no change in cell viability (Figures S9B and S9C). Thus, a series of *in vitro* investigations showed a potent neutralizing activity of the anti-TGF-β1 aptamer.

### Anti-cancer effect of APT-β1-OMe-P in xenograft cancer model mice

**Figure 4:**
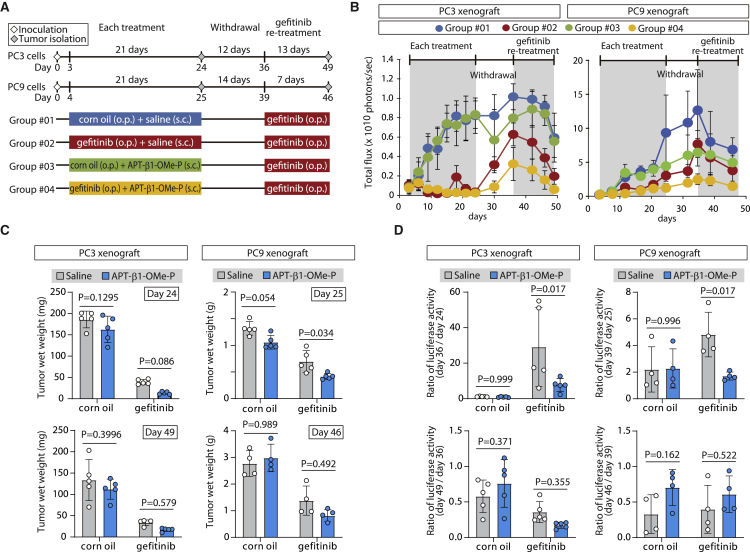
Anti-cancer effect of APT-β1-OMe-P in xenograft cancer model (A) Experimental schedule of xenograft cancer study. Mice with PC3 and PC9 cancer xenografts were treated with APT-β1-OMe-P (10 mg/kg b.w./day) and/or gefitinib (100 mg/kg b.w./day) or their vehicle as a control during indicated periods (details in Materials and methods). (B) *In vivo* luminescent signals of xenografts in each treatment group are shown. Cancer xenografts expressing the *fire luciferase* gene in each treatment group were examined by an IVIS imaging system at indicated days. Until the first sampling day at day 24 or day 25, the *in vivo* imaging was carried out in 10 or 9 mice per group in PC3 or PC9 xenograft, respectively; after the day 24, the monitoring was continued in the remaining 5 or 4 mice per group. (C) Wet weight of tumors in each treatment group is shown. Tumors at day 24 and day 49 in each group in PC3 xenograft (left panel) and at day 25 and day 46 in each group in PC9 xenograft (right panel) were isolated (left panel), and their wet weight was examined (right panel). Statistical differences among treatment groups were examined by two-way ANOVA and then by Sidak multiple comparisons. Data represent the mean ± SD (n = 5 except for at day 46 in PC9; n = 9 at day 46 in PC9 xenograft). (D) Effect of APT-β1-OMe-P on xenografts in drug withdrawal and gefitinib re-treatment periods is shown. To widely evaluate anti-cancer effect of APT-β1-OMe-P on PC3 and PC9 xenografts, the effects of withdrawal period and re-treatment of gefitinib in each treatment group were examined by the ratio of luminescent signals between day 24 and 36 (day 36/day 24), between day 36 and 49 (day 49/day 36) in PC3 xenograft (left panel), between day 25 and day 39 (day 39/day 25), and between day 39 and day 46 (day 46/day 39) in PC9 xenograft (right panel), respectively. As in (C), statistical differences among treatment groups were examined by two-way ANOVA and then by Sidak multiple comparisons. Data represent the mean ± SD (n = 5 except for at day 46 in PC9; n = 9 at day 46 in PC9 xenograft).

To evaluate the *in vivo* effect of TGF-β1 inhibition by APT-β1-OMe-P, a xenograft cancer model was used to assess the anti-tumor effect of APT-β1-OMe-P alone or in combination with the tyrosine kinase inhibitor, gefitinib, before and after the drug withdrawal interval (study design shown in Figure 4A). Gefitinib is a known inhibitor of specific types of cancer, such as PC3 cells and other NSCLCs carrying mutations in exon19 of the *epidermal growth factor receptor* (*EGFR*) gene.^40^ The xenograft model was constructed with PC3 cell, which is not prostate cancer cells but a human NSCLC cell line (details in Materials and methods), and PC9 cells, both of which carry different types of oncogenic mutation in exon19 of the *EGFR* gene^41^^,^^42^ and are closely associated with TGF-β signaling for its progression.^28^ To monitor xenograft tumor *in vivo*, the *firefly luciferase* gene was introduced into PC3 and PC9 cells (details in Materials and methods). Albeit a rough plan, we designed treatment schedule to broadly examine effect of the aptamer in several treatment situations (Figure 4A). Firstly, the effect of TGF-β1 blockade was examined by mono-treatment with APT-β1-OMe-P and combination treatment with APT-β1-OMe-P/gefitinib until day 24 and day 25 in PC3 and PC9 xenograft, respectively, before drug withdrawal (Figures 4B and 4C, upper panel, and S10–S12; Tables S1 and S2). The data clearly indicated a significant inhibition of tumor growth by gefitinib, but not by APT-β1-OMe-P, in mono-treatment. On the other hand, APT-β1-OMe-P in combination therapy for PC9 xenograft significantly inhibited tumor growth compared with mono-treatment with gefitinib (p = 0.034). Meanwhile, there appeared no appreciable difference between gefitinib mono-treatment and combination treatment with APT-β1-OMe-P before drug withdrawal as judged by the luminescent signals derived from each xenograft and the wet weight of isolated tumors from each group (5 out of 10 or 9 mice per group in PC3 or PC9 xenograft, respectively).

However, upon withdrawal of drugs at day 24 in PC3 and at day 25 in PC9 xenograft, the ratio of increased luminescent signals in combination treatment group during the withdrawal period (from days 24 to 36 in PC3 and days 25 to 39 in PC9 xenograft) was significantly suppressed (p = 0.017 in PC3; p = 0.017 in PC9) compared with the gefitinib-only treatment group (Figures 4D, upper panel, S10, and S11; Tables S3 and S4). Thus, the attenuated repopulation of both PC3 and PC9 cells in drug-free period can be interpreted as indicating a specific inhibitory action of APT-β1-OMe-P before drug withdrawal. Upon re-treatment with gefitinib at day 36, PC3 and PC9 growth was inhibited again, generating reduced luminescence signals. The ratio of luminescence signals during days 36–49 in PC3 and days 39–46 in PC9 with or without the preceding treatment with APT-β1-OMe-P was statistically unchanged, showing that TGF-β1 inhibition has no or little effect on re-treatment with anti-cancer drugs after withdrawal (Figures 4C, lower, and 4D, lower panel; Tables S3 and S4). The findings suggest that TGF-β1-specific inhibition may serve as an effective option for combination cancer therapy with certain types of drugs, such as gefitinib, to prevent relapsing after drug withdrawal.

## Discussion

TGF-β has been gaining attention as a therapeutic target in cancer, but the effect of TGF-β1-specific inhibition remains fully elusive due to the limited availability of specific inhibitors. In the present study, we developed anti-TGF-β1 aptamer, APT-β1-OMe-P, and revealed that TGF-β1-specific inhibition in xenograft model enhanced anti-tumor effect of gefitinib on cancer repopulation after withdrawal in combination therapy.

APT-β1-OMe-P binds to human and mouse TGF-β1 proteins with high affinity and specificity and prevents TGF-β1 binding to its receptors, blocking the downstream signaling *in vitro* and *in vivo* (Figures 3B, S13, and S14). The present study revealed that APT-β1-OMe-P alone cannot prevent tumor growth in the mouse xenograft model with PC3 and PC9 cells, human NSCLC cell lines. However, in combination treatment with gefitinib, APT-β1-OMe-P enhanced the tumor-suppressive effect of gefitinib on cancer repopulation after drug withdrawal. In addition, APT-β1-OMe-P significantly repressed PC9 xenograft at day 25, but not PC3 xenograft at day 24; the difference may depend on cell type and dose of gefitinib, which may be so high that aptamer effect was masked. Thus, these results indicated that inhibition of TGF-β1 may be an effective option in combination therapy for cancer. While we found the positive effects of the aptamer in combination treatment, the molecular mechanism remains unknown. One might speculate that drug efflux may be suppressed by TGF-β1 inhibition because the expression of ATP-binding cassette transporters, such as ABCC10, emitting gefitinib from cells increased by TGF-β (Figures S14 and S15),^43^, ^44^, ^45^, ^46^ thereby suggesting retention and prolonged effect of gefitinib in NSCLC. Another option might include tumor microenvironments involving TGF-β1. Recent studies have demonstrated the essential function of tumor microenvironments in regulating the oncogenic activities of TGF-β and its stimulation of metastatic progression during mammary tumorigenesis.^47^ Nevertheless, there are other possibilities to explain for the action mechanism as considering multiple functions of TGF-β1. Recently, it has been reported that anti-cancer effects of programmed cell death-1 (PD-1) and its ligand (PD-L1) blockade therapy were enhanced by the selective inhibition of TGF-β1 signaling using antibodies, such as anti-GARP:TGF-β1 (ABBV-151) and anti-latent TGF-β1 (SRK-181) antibodies.^24^, ^25^, ^26^ Regarding other aptamer approaches targeting TGF-β receptors,^48^^,^^49^ isolated aptamers so far appeared yet to be optimized, and hence, no *in vivo* evaluation was carried out, thereby making it difficult to deeply discuss its therapeutic effects at present.

Our data showed that TGF-β1 upregulated phosphorylation of SMAD2, even at the low dose of 80 pM (Figures 2 and S3). Thus, high-affinity and neutralizing activity is required for medical agents targeting active TGF-β1, and APT-β1-OMe-P may have them (Figures S1 and S2), although we need to consider various things, such as difference of responsivity to TGF-β1 in each cell type, abundance ratio of active and inactive form of TGF-β1 in microenvironments, and delivery efficacy of medical agent (Figures S1 and S13), Since TGF-β2 and -β3 play important functions in the cardiovascular system in mice,^14^, ^15^, ^16^ albeit with no definitive and direct evidence, there is a possibility that the TGF-β1-specific inhibition may have few adverse effects compared with pan-TGF-β inhibition. These reports may support a good safety profile of APT-β1-OMe-P because the aptamer has high specificity to TGF-β1, limited antigenicity, and irrelevance to antibody-dependent cellular cytotoxicity; therefore, APT-β1-OMe-P may be a promising agent for combination cancer therapy with few side effects. Given that TGF-β1 is closely involved in metastasis and resistance to chemotherapy, further studies are needed to evaluate the effect of TGF-β1-specific inhibition on various deteriorating events of NSCLC or other cancers using different animal models to examine involvement of immune system, such as syngeneic model, rescue experiments with agonists, and negative control sequence for more convincing study. Although our result may be a small advance in cancer biology, the current study suggests that active TGF-β1 inhibitory strategies without affecting the other isoforms and its latent form may serve as relapse prevention strategy through suppression of TGF-β1-induced expression of EMT-related genes and ABC transporter in certain types of combination therapies for NSCLCs. Taken together with previous studies, our findings reinforce the notion that inhibition of TGF-β1 might not affect the proliferation and viability of NSCLC effectively in monotherapy but may serve as a supportive agent for other anti-cancer drugs in combination therapy. The highly specific and strong affinity of APT-β1-OMe-P to TGF-β1 should promote us to further assessment in both neoplastic and non-neoplastic applications.

## Materials and methods

### Aptamer selection and manipulation of aptamer

To construct RNA library, single-stranded DNA (ssDNA) library was purchased from GeneDesign (Ibaraki, Osaka, Japan), and its sequence is as follows: 5′-GAC TGA CGT CGC ACT [N35] AGC TCC AAG TTC TCC C-3ʹ (where N35 represents 35-nt random sequence). Primer sequences for PCR amplification, reverse transcription, and *in vitro* transcription (IVT) were indicated as follows: forward, 5′-TAATACGACTCACTATAGGGAGAACTTGGAGCT-3′; reverse, 5′-GACTGACGTCGCACT-3′. T7 promoter sequence is underlined in forward primer. Double-strand DNA library was synthesized using the ssDNA library, forward primer containing T7 promoter, and ExTaq DNA polymerase (TaKaRa Bio, Shiga, Japan). The synthetic double-stranded DNAs (dsDNAs) were then subjected to IVT using 2′-fluoro-CTP, 2′-fluoro-UTP, ATP, and guanosine triphosphate (GTP). To efficiently incorporate 2′-fluoro pyrimidines in aptamer sequences, Y639F T7 RNA polymerase was used in IVT process.^50^

For aptamer selection, SELEX against recombinant human TGF-β1 (PeproTech, NJ, USA) was carried out as previously described.^51^ Briefly, constructed RNA library was subjected to a common aptamer affinity selection against TGF-β1 recombinant protein, which was immobilized to NHS-activated Sepharose 4 Fast Flow (GE Health Care Life Sciences, MA, USA). In every selection round except for first round, Sepharose beads were subjected to a negative selection, i.e., subtraction process. After selections, candidate sequences were chosen based on the SPR analysis and cell base assay using SMAD promoter assay described below.^32^

As for manipulations of aptamers, the lead aptamer was shortened to 33 nt without losing activity and modified with 2′-O-methyl substitutions to confer the resistance to nucleases. 2′-O-methyl substitutions were carried out comprehensively, and 20 sequences were tested based on SPR analysis and SMAD promoter assay (patent application no. WO/2021/006,305).^32^ The aptamer is 33 nt in length and contains 2′-fluoro modified bases at five positions, 2′-O-methly modified bases at 18 positions, and 2′-hydroxy unmodified bases at 10 positions. The aptamer was chemically synthesized (Gene Design) and examined by several experiments. As for animal experiments, the aptamer was further modified by 40-kDa PEG (JenKem Technology, TX, USA) and inverted dT at 5′-and-3′ end, respectively.^32^ The sequence is as follows: G(M)GC(F)A(M)U(F)AAG(M)G(M)G(M)A(M)G(M)GGGA(M)G(M)AC(F)U(F)U(F)GU(M)G(M)G(M)A(M)G(M)GGC(M)A(M)A(M)G(M), where (M) and (F) represent 2′-O-methyl and 2′-fluoro modified bases, respectively.

### SPR analysis

SPR analyses were carried as previously described using BIAcore T200 instrument (GE Health Care Life Sciences).^51^^,^^52^

To examine binding ability of aptamers to recombinant TGF-βs, 60–140 resonance units (RUs) of aptamer labeled with biotin at 5′ end was immobilized onto streptavidin (SA) sensor chip. Running buffer was SELEX buffer (145 mM NaCl, 5.4 mM KCl, 0.8 mM MgCl2, 1.8 mM CaCl2, and 20 mM Tris-HCl [pH 7.6]) supplemented with 0.05% Tween 20. After immobilization of aptamer, the following proteins at final concentration of 500 nM were injected for 120 s: human TGF-β1 (PeproTech), mouse TGF-β1 (R&D systems, MN, USA), human TGF-β2 (PeproTech), human TGF-β3 (PeproTech), human latent TGF-β1 (R&D system), and human alpha-Thrombin (Haematologic Technologies, VT, USA) as a negative control. To regenerate sensor chips, solution consisting of 2 M NaCl and 10 mM NaOH was injected for 30 s in regeneration process.

To examine inhibition effect of aptamers on ligand-receptor interaction, about 1,500 RUs of protein A was immobilized onto CM5 sensor chip by amino coupling. Running buffer was the same as in a binding analysis described above. After immobilization of a protein A, recombinant TGF-βRII/Fc fusion protein at a final concentration of 30 nM was injected, resulting in immobilization of the receptor protein about 130 RUs. After injection of the receptor protein, premixtures of TGF-β1 (PeproTech) and aptamers at indicated concentrations were further injected. In regeneration process, 6 M guanidine hydrochloride solution was used.

For estimation of dissociation constant (K_D_ value) of APT-β1-OMe-P, about 600 RUs of recombinant human TGF-β1 (R&D Systems) was immobilized onto CM4 sensor chip by amino coupling. Running buffer was the same as in a binding analysis. After immobilization of TGF-β1, binding ability of the aptamer was analyzed by single cycle kinetics. Briefly, the aptamer at indicated concentrations was sequentially injected in ascending order of concentrations in the same cycle without regeneration.

In addition to estimation of K_D_ value of APT-β1-OMe-P using CM4 sensor chip, K_D_ value was estimated by using 5′ biotinylated APT-β1-OMe-P and SA sensor chip. About 60 RUs of 5′ biotinylated APT-β1-OMe-P was immobilized onto SA sensor chip, and then binding ability of the aptamer was analyzed by single-cycle kinetics. The K_D_ value was estimated using univalent and bivalent curve fitting model by BIAcore T200 Evaluation software (GE Health Care Life Sciences).

### Cell culture

HEK293 cells and A549 cells were obtained from the American Type Culture Collection (CRL-1573 and CCL-185, respectively; Manassas, VA, USA) and grown in DMEM medium (Wako Pure Chemicals Industries, Osaka, Japan) supplemented with 10% fetal bovine serum (Life Technologies, CA, USA) and 100 units/mL penicillin and 100 mg/mL streptomycin (Wako) at 37°C in a 5% CO_2_ humidified chamber. A human NSCLC cell line, PC-3 cells, and PC9 cells were obtained from Health Research Resources Bank (JCRB no. JCRB0077) and RIKEN BRC (RCB4455), respectively, and both cell lines were grown in RPMI-1640 medium (Wako) supplemented with 10% fetal bovine serum, 100 units/mL penicillin, and 100 mg/mL streptomycin (Wako) at 37°C in a 5% CO_2_ humidified chamber.

### SMAD2 promoter assay

HEK293 cells were treated with trypsin, suspended in fresh medium without antibiotics, and seeded onto 6-well culture plates at a cell number of 2 × 10^5^ cells/well. The day after seedings, the pGL4.48[luc2P/SBE/Hygro] vector (2.2 mg; Promega, WI, USA) was diluted with 100 mL Opti-MEM (Life Technologies) and then mixed with 100 μL Opti-MEM containing 6 mL PEI 250,000 (Polysciences, PA, USA) at a concentration of 0.3 g/L. After incubation for 15 min at room temperature, transfection mixtures were added to each well. After 24 h, the cells were detached with trypsin again and re-seeded onto 96-well culture plate at a cell number of 1 × 10^4^ cells/well. After further 1 day incubation, the cells were treated with premixtures of TGF-β1 (PeproTech) and each aptamer or anti-pan-TGF-β antibody (MAB1835, clone no. 1D11, R&D Systems) at indicated concentrations for 3 h, which were diluted with DMEM without serum. The incubated cells were lysed with passive lysis buffer (PLB) (Promega), and the expression levels of luciferases were examined by Dual Luciferase Reporter System (Promega). The luminescent signals were measured with CentroXS^3^ LB960 (Berthold Technologies, Bad Wildbad, Germany).

### Cellular morphological changes

A549, PC3, and PC9 cells were treated with trypsin, suspended in fresh medium, and seeded onto 24-well culture plates at a cell number of 1.5 × 10^4^ cells/well. After 1 day incubation, cells were treated with premixture of TGF-β1 (PeproTech) and each aptamer at indicated concentrations and incubated for 48 h.

### Western blot analysis

HEK293, A549, PC3, and PC9 cells were treated with trypsin, suspended in fresh medium, and seeded onto 24-well culture plates at a cell number of 1.5 × 10^4^ cells/well. Briefly, HEK293, PC3, and PC9 cells were used for examining phosphorylation of SMAD2, and A549, PC3, and PC9 cells were used for examining altered expression of EMT-relevant genes. One day after seeding, cells were treated with premixtures of TGF-β1 (PeproTech) and each aptamer or anti-pan-TGF-β antibody (MAB1835, R&D Systems) at indicated concentrations for 3 h to detect p-SMAD2 in HEK293, PC3, and PC9 cells or for 24 h to detect altered expression of EMT-related proteins and ABCC10 in A549, PC3, and PC9 cells. The treated cells were lysed with 50 mL lysis buffer (20 mM Tris-HCl [pH 7.5], 150 mM NaCl, 1 mM DTT, 1 mM EDTA, and 1% NP-40) containing a 1× protease inhibitor cocktail (Protease Inhibitor Cocktail Tablets; Roche Diagnostics, Basel, Switzerland) per well. Cell lysates were mixed with 5× sample buffer, boiled for 5 min, and then separated by SDS-PAGE on 10% polyacrylamide gels. The proteins of interest were visualized using antibodies described below and Immobilon Western Chemiluminescent HRP Substrate (Millipore). The primary and secondary antibodies used in western blot analysis were as follows: rabbit monoclonal anti-Smad2 (D43B4) XP antibody (Cell Signaling Technology, Danvers, MA, USA), rabbit monoclonal anti-phospho-Smad2 (Ser465/467) (138D4) antibody (Cell Signaling Technology), rabbit monoclonal anti-vimentin (D21H3) XP antibody (Cell Signaling Technology), rabbit monoclonal anti-Snail (C15D3) antibody (Cell Signaling Technology), rabbit monoclonal anti-N-cadherin (D4R1H) XP antibody (Cell Signaling Technology), rabbit monoclonal anti-E-cadherin (24E10) antibody (Cell Signaling Technology), rabbit polyclonal anti-collagen alpha 1(I) antibody (Abcam, Cambridge, UK), rabbit monoclonal anti-collagen alpha 1(I) (E8F4L) XP antibody (Cell Signaling Technology), mouse polyclonal anti-ABCC10 antibody (ab69296) (Abcam), and mouse monoclonal anti-alpha tubulin antibody (Millipore), horseradish peroxidase-conjugated anti-rabbit or anti-mouse immunoglobulin G (IgG) antibody (Jackson ImmunoResearch Laboratories, West Grove, PA, USA).

For *in vivo* experiments, isolated tumors were homogenized with radioimmunoprecipitation assay (RIPA) buffer containing a 1× protease inhibitor cocktail (Protease Inhibitor Cocktail Tablets; Roche), and then subsequent processes in western blotting were performed as in the experiment using cell lysate described above.

### Gene expression analysis

A549 cells were treated with trypsin, suspended in fresh medium, and seeded onto 96-well culture plate at a cell number of 1 × 10^4^ cells/well. After overnight incubation, culture medium was replaced with reduced serum DMEM containing 0.1% FBS. The day after medium replacement, cells were treated with premixtures of TGF-β1 (PeproTech) and each aptamer or anti-pan-TGF-β antibody (R&D Systems) at indicated concentrations for 24 h, which were diluted with DMEM without serum. The treated cells were washed with phosphate-buffered saline (PBS), lysed, and then subjected to cDNA synthesis using a SuperPrep Cell Lysis & RT Kit for qPCR (Toyobo, Osaka, Japan) according to manufacturer’s protocol. The synthetic cDNAs were examined by real-time PCR (qPCR) using an AB 7300 Real-Time PCR System (Applied Biosystems) and a TaqMan Universal PCR Master Mix. TaqMan probes for human COL1A1, VIM, and CDH1 were purchased from Life Technologies.

### Xenograft model and administration of gefitinib and aptamer

To monitor tumor growth by luminescent signals, the firefly luciferase gene was introduced into PC3 and PC9 cells using a lentiviral vector, which was constructed by insertion of entire coding sequence of the *firefly luciferase* gene into a multicloning site of pHAGE-CMV-MCS-IZsGreen vector using an In-Fusion HD Cloning Kit (TaKaRa Bio). The cells expressing the luciferase gene were isolated by a BD FACSAria III cell sorter based on the fluorescent signal of GFP that fused with the luciferase gene via IRES. As for PC9 cells, single cell was isolated by fluorescence-activated cell sorting (FACS) and cloned. After generation of luciferase-positive PC3 (PC3/luc) and PC9 (PC9/luc) cell lines, the PC3/luc and PC9/luc cells were treated with trypsin and resuspended in PBS containing 50% Matrigel (BD Biosciences, San Jose, CA, USA) at a final concentration of 1 × 10^7^ cells/mL and 10 × 107 cells/mL, respectively; 200 μL of cell suspension (≈2 × 10^6^ cells for PC3 and ≈5 × 10^6^ cells for PC9) were injected subcutaneously into the right flank of individual non-obese diabetic (NOD)-severe combined immunodeficiency (SCID) mice anesthetized by isoflurane (Pfizer, NY, USA).

Xenograft tumors were monitored with an IVIS imaging system (Xenogen, Alameda, CA, USA) according to the manufacturer’s instructions at indicated days. Briefly, photographic images of the luminescent signal intensities were taken 10 min after injection of D-luciferin (75 mg/kg body weight [b.w.]), and the images were analyzed using a Living Imaging software (Xenogen).

As for treatments to xenograft cancer model, the mice were administered by gefitinib (100 mg/kg/day, 200 μL oral administration [p.o.]) and/or aptamer (10 mg/kg b.w./day, 200 μL subcutaneous injection [s.c.]) as mono- or combination therapy from day 3 to day 24 and day 4 to day 25 after injection of PC3/luc cells and PC9/luc cells, respectively. Likewise, as control treatments, administration of corn oil (200 μL/day, p.o.) and/or saline (200 μL/day, s.c.) were carried out to each group as necessary.

From day 24 in PC3 xenograft and day 25 in PC9 xenograft, all treatment was stopped for examining effects of repopulation of cancer cells for 12 days and 14 days in PC3 and PC9 xenograft, respectively. After drug withdrawal, gefitinib treatment to all groups was started from day 36 to day 49 in PC3 xenograft and from day 39 to day 46 in PC9 xenograft, for examining anti-cancer effect of gefitinib treatment on repopulating cancer cells in each treatment.

NOD-SCID mice were obtained from Charles River Japan. Mice were housed, fed, and maintained under special pathogen-free conditions according to the animal care guidelines of the Institute of Medical Science, The University of Tokyo (IMSUT). Animal experiments were approved by the Committee on the Ethics of Animal Experiments of the IMSUT and were performed in accordance with the Guidelines for Animal Experiments of the IMSUT.

### ELOSA assay for APT-β1-OMe-P

To confirm delivery of APT-β1-OMe-P to tumor, PC9-luc cells (5 × 10^6^ cells) were inoculated into left flank of NOD/SCID mice. Thirty-two days after the inoculation, aptamer APT-β1-OMe-P at a dose of 10 mg/kg or saline as a vehicle control (n = 2/group) was injected to the mice bearing tumor. One day after the aptamer injection, concentrations of the aptamer in several organs (brain, kidney, and liver), plasma, and tumor were examined by enzyme-linked oligosorbent assay (ELOSA).

ELOSA was basically performed according to the previous report.^53^ Briefly, APT-β1-OMe-P concentration in tumor, plasma, and several tissues (brain, kidney, and liver) was measured with a hybridization-based dual-capture pseudo-ELISA method using detection probe attached with FAM and capture probe attached with amino linker C6. Mediating horseradish peroxidase (HRP)-conjugated anti-fluorescein isothiocyanate (FITC) antibody, standard ELISA procedure was carried out with common TMB substrate. Tissue concentration of APT-β1-OMe-P was calculated with GraphPad Prism 8.

### Statistical analysis

Statistical analysis was performed by GraphPad Prism 8 (GraphPad Software, CA, USA). Curve fitting with nonlinear regression and the analysis of EC50 and IC50 were also performed by the software (Figures 2, 3A, and S6). For Figures 3B, S14, and S15, the data were analyzed by one-way analysis of variance (ANOVA) followed by Turkey-Kramer test. As for Figures 4C and 4D, the obtained data were analyzed by two-way ANOVA followed by Sidak’s multiple comparisons. For Figure S4, the data were analyzed by one-way ANOVA followed by Dunnett’s multiple tests. For all statistical analyses, the alpha value was set at 0.05.

## Data Availability

All data associated with this study are present in the paper, the supplemental information, and Nakamura et al.^32^

## Supplemental information

Document S1. Figures S1–S15 and Tables S1–S4

Document S2. Article plus supplemental information
