# Supplementary material for: Anti-TGF-β1 aptamer enhances therapeutic effect of tyrosine kinase inhibitor, gefitinib, on non-small cell lung cancer in xenograft model
Source: Mol Ther Nucleic Acids. 2022 Jun 29;29:969–78. doi: 10.1016/j.omtn.2022.06.001 (PMC9481871; doi:10.1016/j.omtn.2022.06.001)
Supplement: Document S1. Figures S1–S15 and Tables S1–S4 [file mmc1.pdf]

OMTN, Volume *29*

Supplemental information

Anti-TGF-β1 aptamer enhances therapeutic effect of tyrosine kinase inhibitor, gefitinib, on non-small cell lung cancer in xenograft model

Masaki Takahashi, Yoshifumi Hashimoto, and Yoshikazu Nakamura

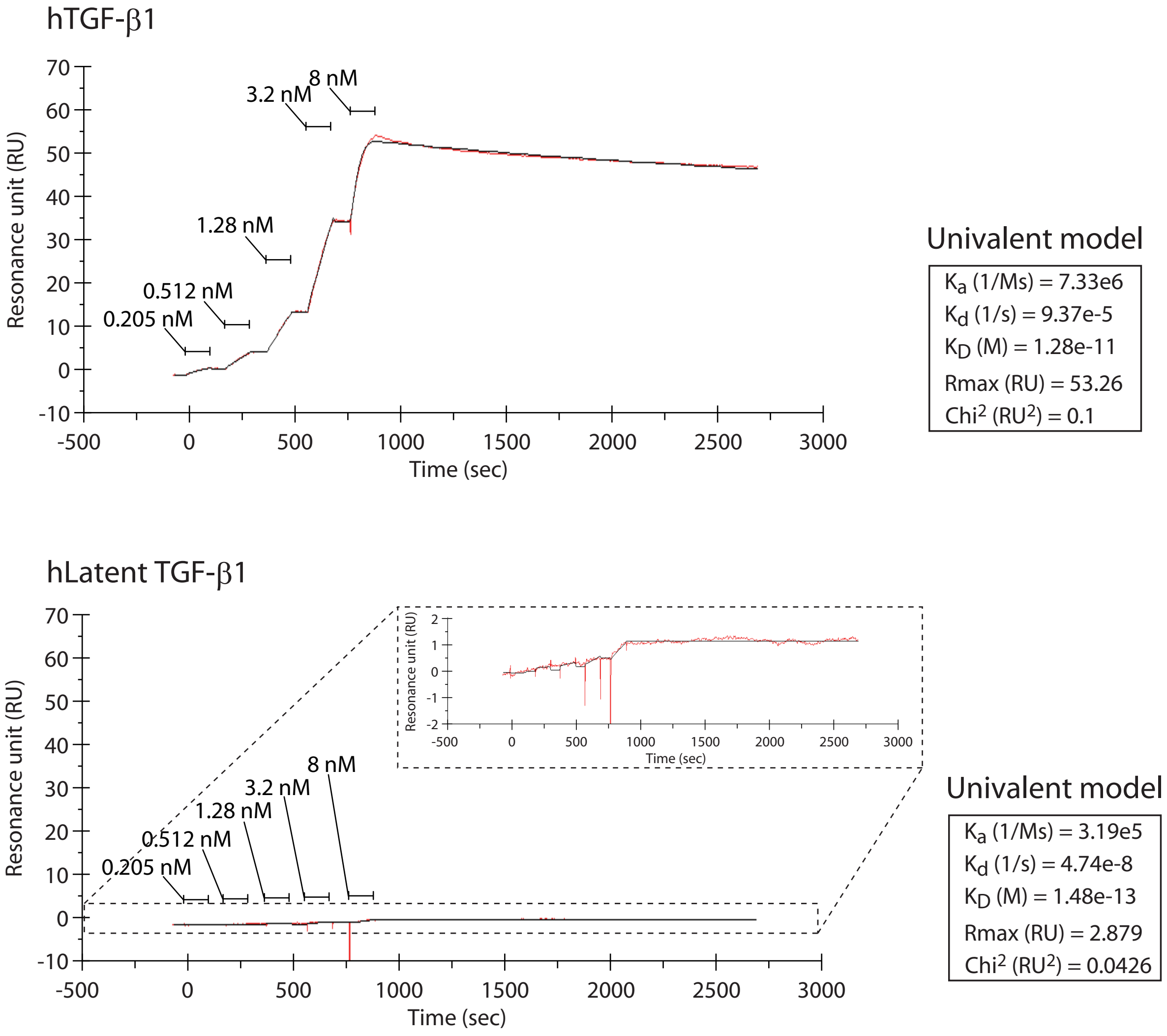

Fig. S1. Binding ability of APT-β1-OMe-P to human latent TGF-β1 protein. Binding ability of immobilized APT-β 1-OMe-P to human TGF-β1 (upper panel) and latent TGF-β1 protein (lower panel). Binding ability of biotinylated APT-β 1-OMe-P, which were immobilized to a SA sensor chip, to each form of human TGF-β1 protein were examined by a single-cycle kinetics with a BIAcore system, and the estimated parameters (Ka, Kd, KD, Rmax [RU], and Chi^2 [RU^2]) that were analyzed by univalent fitting model were shown. The result showed that APT-β1-OMe-P has no or little binding ability to latent type TGF-β1 protein.

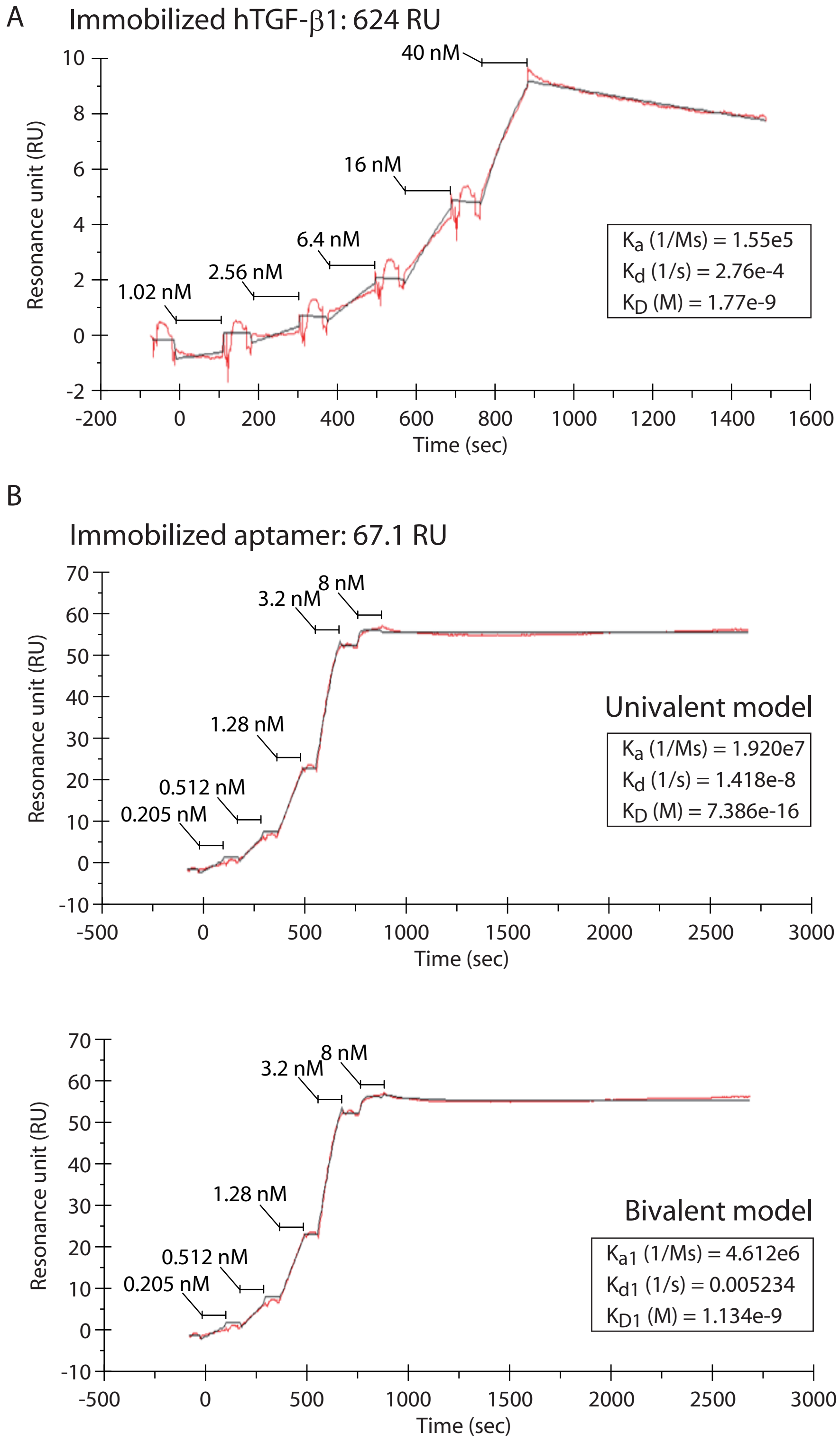

Fig. S2. Estimation of the dissociation constant of APT-β1-OMe-P by SPR analysis. (**A**) Binding ability of APT-β1-OMe-P to immobilized TGF-β1 protein. Binding ability of various amounts of APT-β1-OMe-P to human TGF-β1 immobilized to a sensor chip were examined by a single-cycle kinetics with a BIAcore system, and the estimated parameters (K_a, K_d, and K_D) that analyzed by univalent fitting model were shown. In this case, it was not certain that the value was accurate because the result unexpectedly showed low binding response with distorted sensorgrams considering the amount of immobilized protein. This might be due to the loss of native protein structure and/or aptamer binding site(s) in the immobilization process. (**B**) Binding ability of immobilized APT-β1-OMe-P to TGF-β1 protein. Binding ability of biotinylated APT-β1-OMe-P, which were immobilized to a SA sensor chip, to human TGF-β1 were examined by a single-cycle kinetics with a BIAcore system, and the estimated parameters (K_a, K_d, and K_D) that were analyzed by univalent fitting model (upper panel; K_D value = 738 attomolar) and bivalent fitting model (lower panel; K_{D1} value = 1.13 nanomolar), respectively, were shown, because the recombinant TGF-β1 is expected to form homodimer. Since the actual binding manner of the aptamer on the sensor chip was unknown, it was not clear that either value is correct. In addition, in this assay, the analytical software cautioned that the kinetics constant K_d value was outside of the limitation of the instrument due to the extremely slow dissociation rate, suggesting the difficulty of determining accurate K_D values of the aptamer.

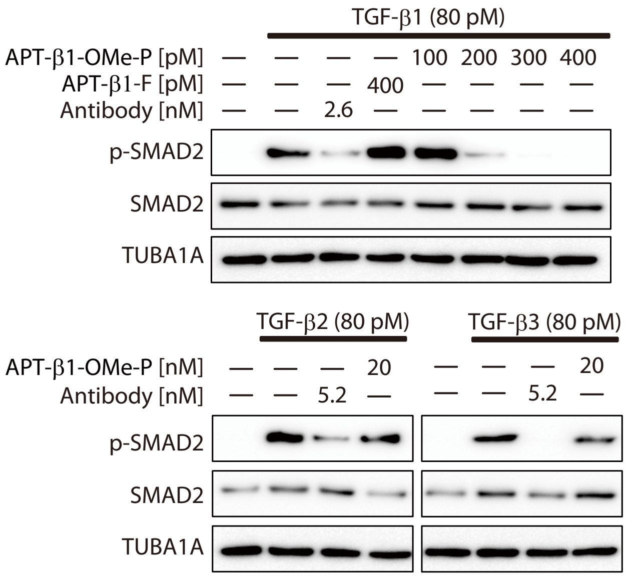

Supplementary Fig. S3 Inhibitory effect of aptamers on TGF-β1-induced phosphorylation of SMAD2. HEK293 cells were treated with or without three TGF-β isoforms (80 pM) in the presence or absence of aptamers at indicated concentrations for 3 h, and phosphorylation levels of SMAD2 was examined by western blot analysis. APT-β1-F was used as a negative control because the aptamer resulted in little inhibition activity in the luciferase assay. Antibody was an anti-pan-TGF-β monoclonal antibody possessing neutralizing activity. The expression levels of TUBA1A (tubulin alpha 1a) were examined as a loading control.

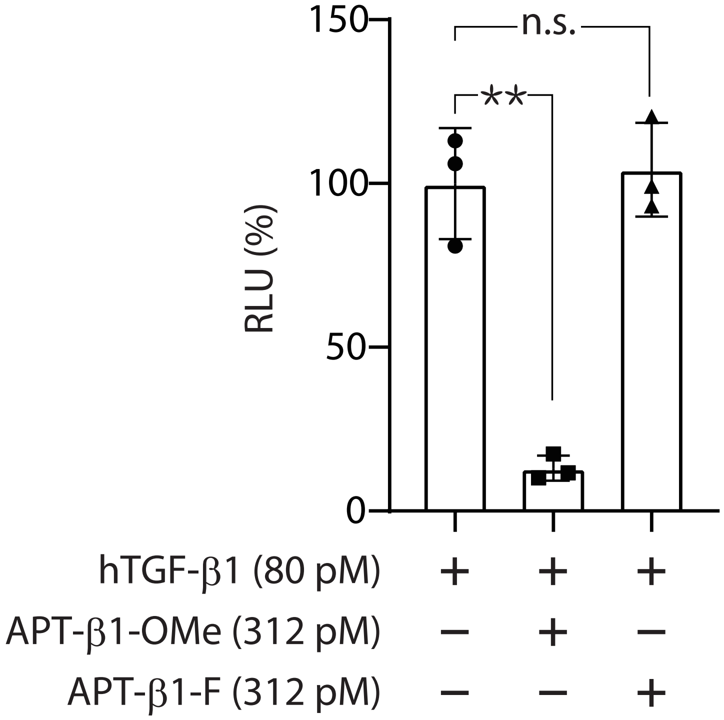

Fig. S4. Inhibition of hTGF-β1-induced expression of luciferase reporter by aptamers. As in Fig.2, HEK293 cells were transfected with the luciferase reporter gene driven by activated (phosphorylated) SMAD2, and then the cells were treated with human TGF-β1 (80 pM) in the presence or absence of indicated aptamers. APT-β1-F arose from the sequence optimization process of APT-β1 (i.e. APT-β1 derivative) and resulted in little inhibitory potential compared to that of APT-β1-OMe. The values were expressed as relative luminescent units (RLU shown in %) to the 80 pM hTGF-β1 level without aptamer after subtraction of basal LU in control cells without treatment. Data represent the mean ± s.d. (n=3). Statistical differences were examined by analysis of variance (ANOVA) and then by Dunnett’ s multiple comparisons. ** $P<0.01$, n.s.; not significant, between indicated pairs.

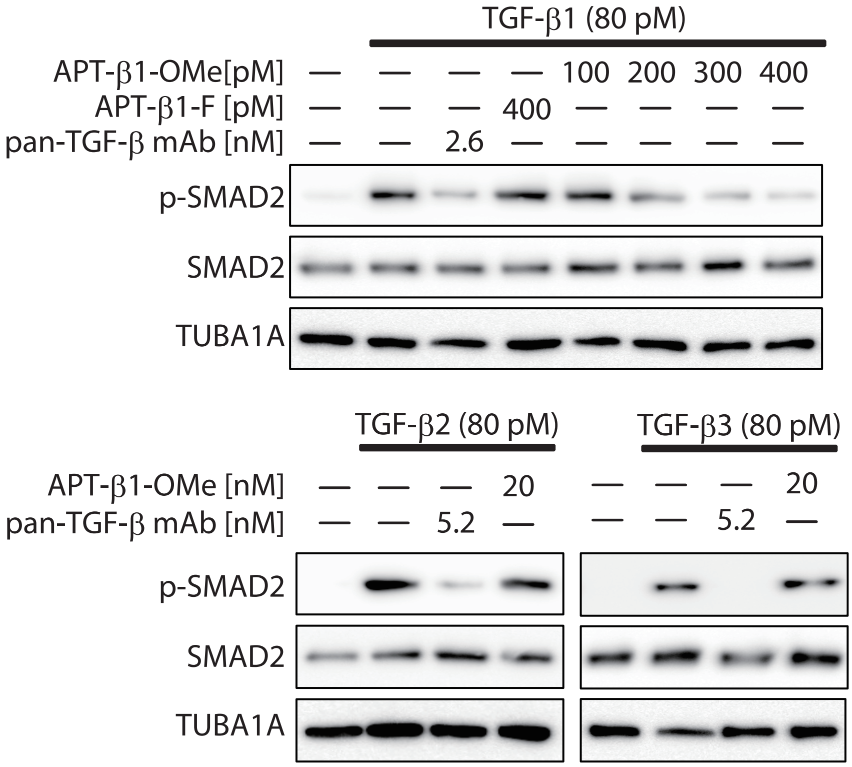

Fig. S5. Inhibition of TGF-β1-induced phosphorylation of SMAD2 by APT-β1-OMe. As in Fig. S3, the effect of APT-β1-OMe on TGF-β isoforms-induced phosphorylation of SMAD2 in HEK293 cells was examined. Inhibitory potential of anti-pan-TGF-β antibody and APT-β1-F as positive and negative control, respectively, were also examined.

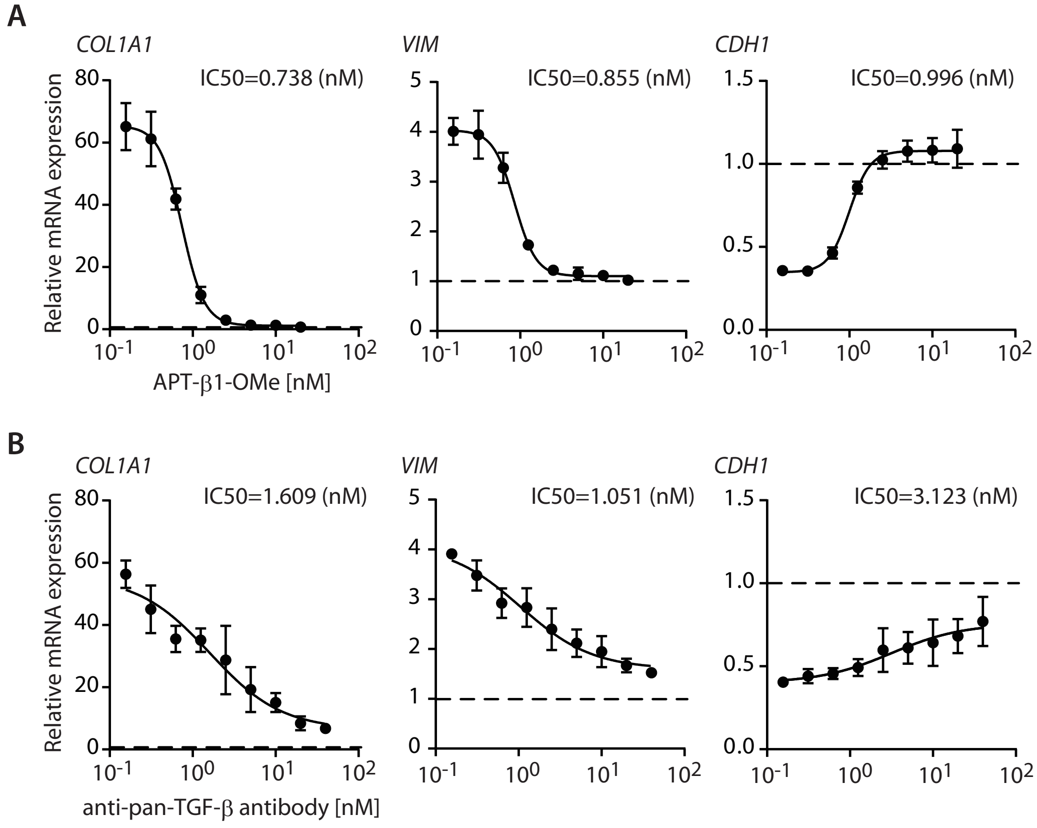

Fig. S6. Inhibitory effect of APT-β1-OMe on alterations in the mRNA expression of TGF-β1-induced EMT-related genes in A549 cells. (**A**) As in Fig.3A, when A549 cells were treated with or without 0.4 nM TGF-β1 in the presence or absence of APT-β1-OMe, the effect of APT-β1-OMe on changes in the mRNA expression of TGF-β1-induced EMT-related genes was examined. (**B**) Anti-pan-TGF-β antibody was also subjected to the same analysis. The expression levels of those genes were normalized by the expression levels of GAPDH, and then the normalized values of each gene in the various treated cells were expressed as relative expression levels to those in the control cells without any treatments as one, which is indicated by a dashed line. Data represent the mean ± s.d. (n=3). The IC50 values of APT-β1-OMe and anti-pan-TGF-β1 antibody in the mRNA expression analysis were shown in each graph.

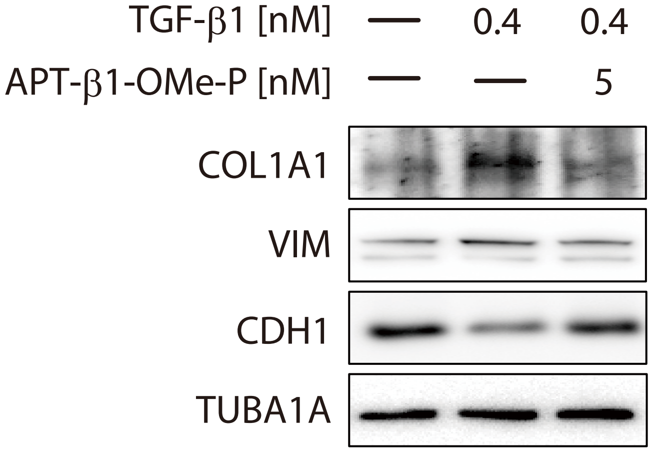

Supplementary Fig. S7 Altered protein expression levels of TGF-β1-induced EMT-related genes by APT-β1-OMe-P. As in Fig. 3A, A549 cells as a major TGF-β1-responsive cells were treated with or without TGF-β1 (0.4 nM) in the presence or absence of APT-β1-OMe-P (5 nM) for 24 h, and the protein expression levels of indicated three genes were examined by western blot analysis. The expression levels of TUBA1A were examined as a loading control.

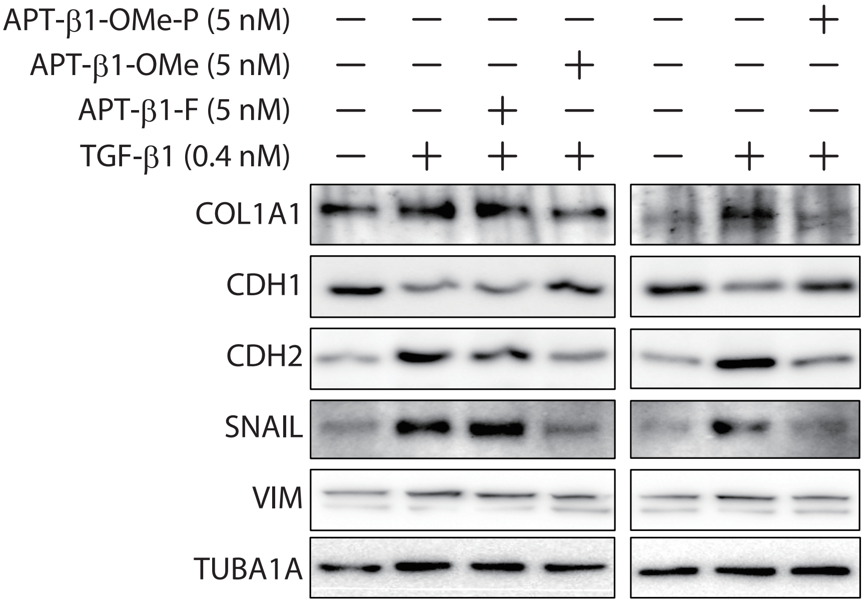

Fig. S8. Inhibitory effect of APT-β1-OMe on alteration in the protein expression of TGF-β1-induced EMT-related genes in A549 cells. As in Fig.S7, when A549 cells were treated with or without 0.4 nM TGF-β1 in the presence or absence of APT-β1-OMe, the effect of APT-β1-OMe on changes in the protein expression of TGF-β1-induced EMT-related genes was examined. APT-β1-F was used as a negative control. The protein expression levels of SNAIL and CDH2 were also analyzed in addition to COL1A1, CDH1, and VIM, as TGF-β1-responsive EMT-related genes.

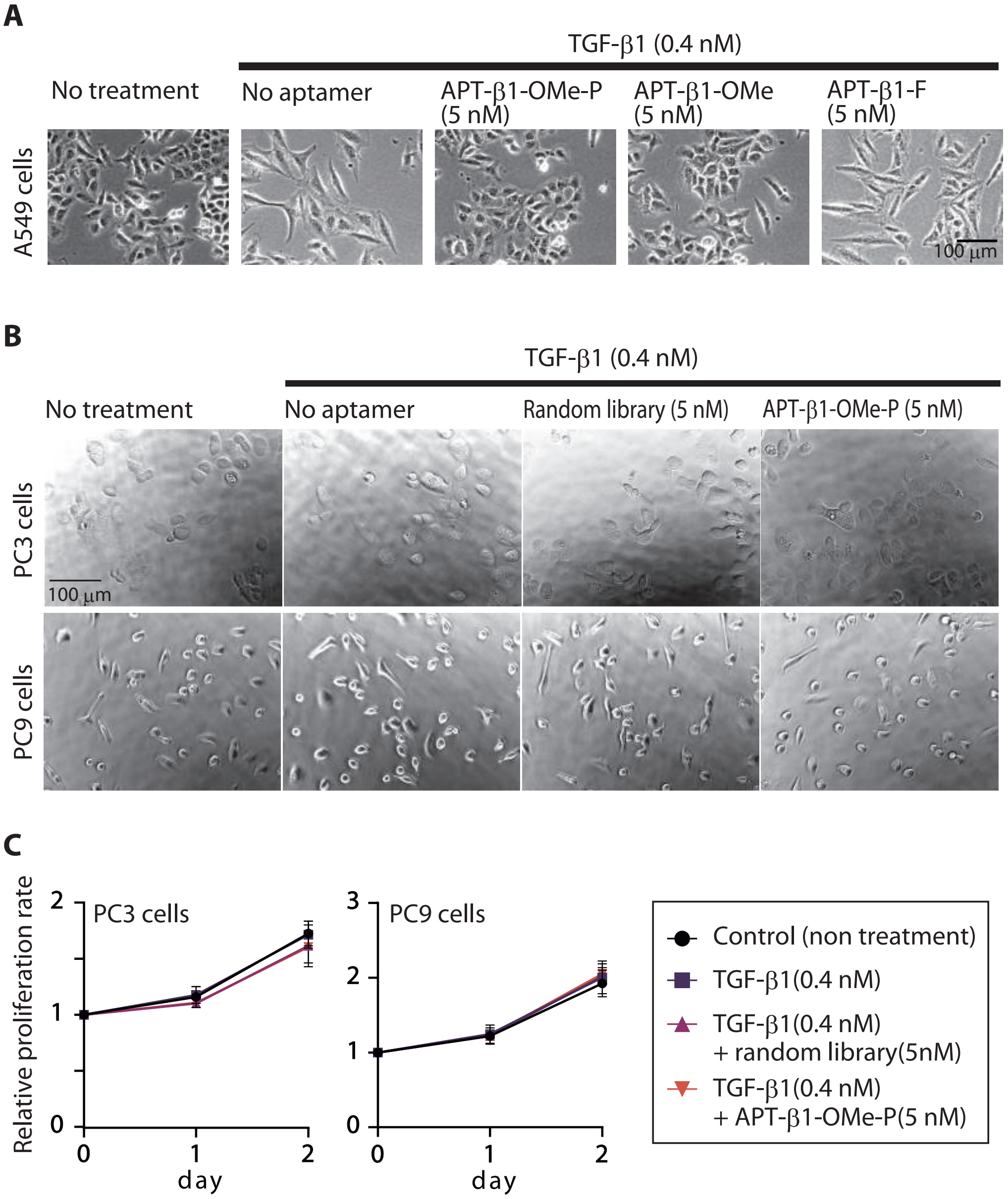

Fig. S9. Effect of APT-β1-OMe(-P) on TGF-β1-induced morphological changes and on cell viability in lung cancer cells. (**A**) Effect of APT-β1-OMe with or without PEG on TGF-β1-induced morphological changes in A549 cells and (**B**) PC3 and PC9 cells, NSCLCs. When A549 cells were treated with or without 0.4 nM TGF-β1 in the presence or absence of APT-β1-OMe (5 nM) and APT-β1-OMe-P (5 nM) for 48 h, the effect of APT-β1-OMe and APT-β1-OMe-P on TGF-β 1-induced morphological changes was examined in A549 cells (**A**) and NSCLCs, PC3 and PC9 cells (**B**). APT-β1-F and random library were used as negative controls in A549 cells and NSCLCs, respectiely. (**C**) Effect of TGF-β1 on cell viability of PC3 and PC9 cells, NSCLCs. When PC3 and PC9 cells were treated with or without 0.4 nM TGF-β1 in the presence or absence of APT-β1-OMe-P (5 nM) and random library (5 nM) as a negative control for 0, 1, and 2 days, the effects of TGF-β1 treatment on cell viability in PC3 and PC9 cells were examined. However, significant changes were not observed between control and each treatment in each cell line. The values of cell viability in those cells were normalized by the values of cell viability in each cell line without treatment at day 0 as one. Data represent the mean ± s.d. (n=3).

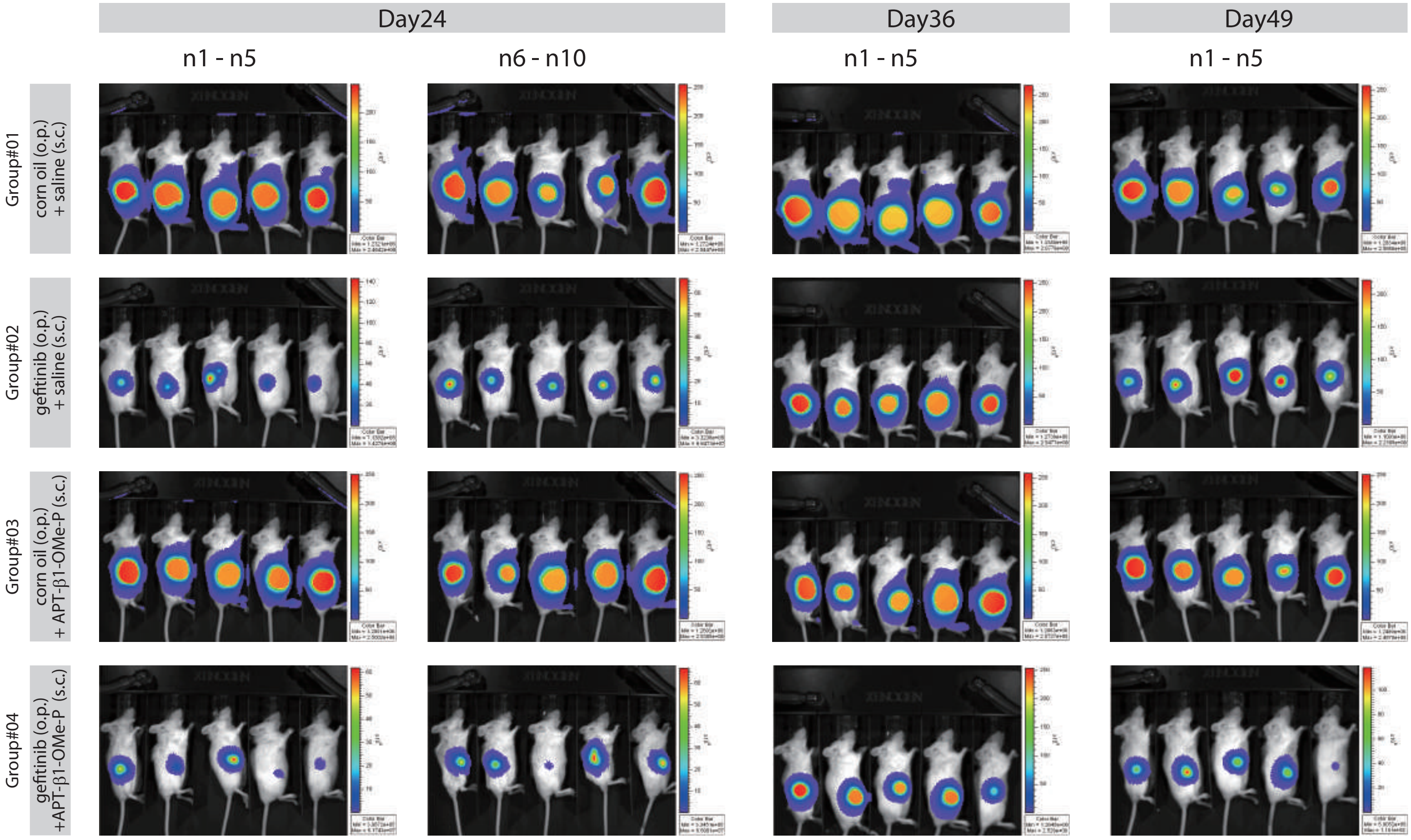

Fig. S10. In vivo luminescent images of PC3 xenograft mice in each treatment group. The luminescent images of PC3 xenograft mice in each treatment group at day 24, 36, and 49 were shown. As described in Methods, ten mice per group were examined until day 24 at when the first sampling was carried out, and thereafter, the monitoring using *in vivo* imaging continued with the remaining five mice per group, as shown in Fig. 4B. The images of isolated tumors from the mice in each group were presented in Fig.S12. The luminescent images were analyzed by IVIS imaging system and used for Fig.4B and 4D.

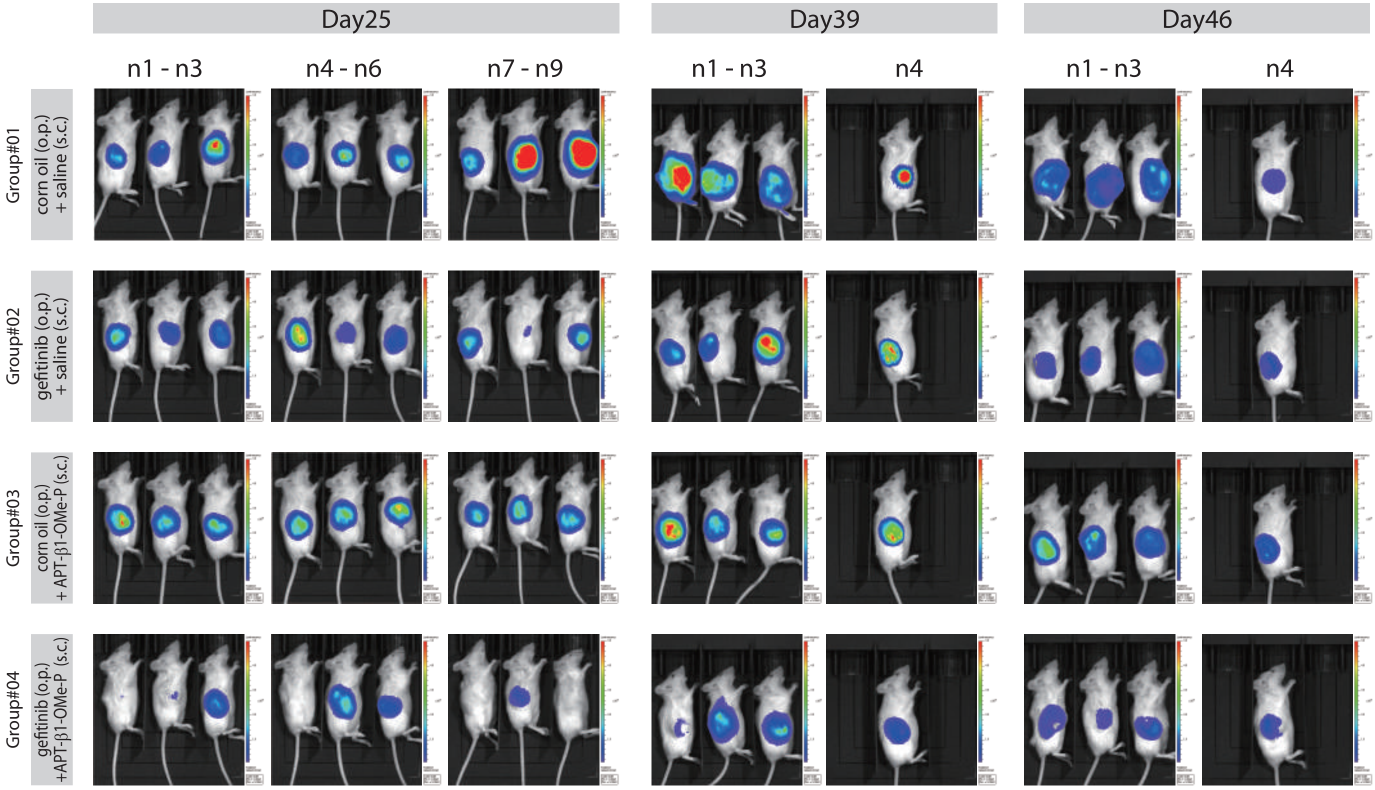

Fig. S11. In vivo luminescent images of PC9 xenograft mice in each treatment group. As in figure S10, the luminescent images of PC9 xenograft mice in each treatment group at day 25, 39, and 46 were shown. Nine mice per group were examined until day 25 at when the first sampling was carried out, and thereafter, the monitoring using in vivo imaging continued with the remaining five mice per group, as shown in Fig. 4B. The images of isolated tumors from the mice in each group were presented in Fig.S12. The luminescent images were analyzed by IVIS imaging system and used for Fig.4B and 4D.

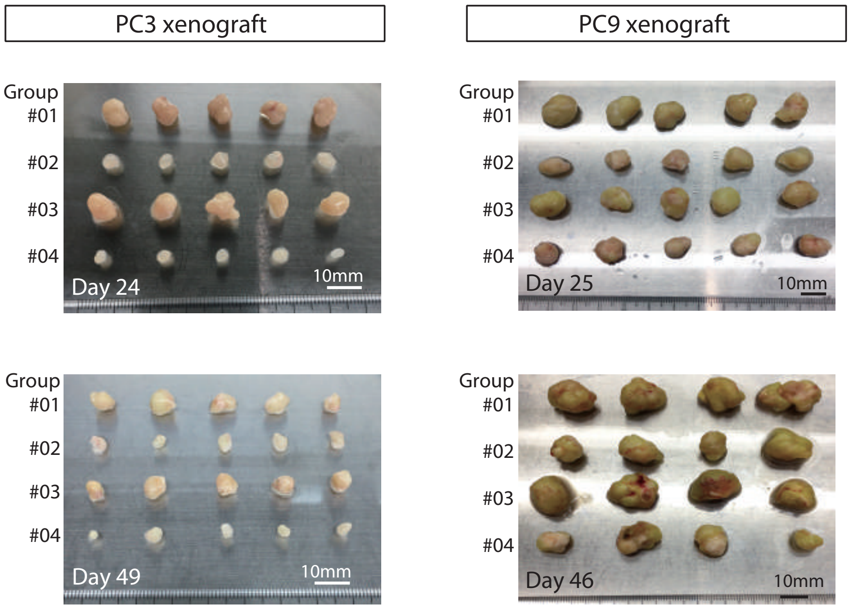

Fig. S12. Images of isolated PC3 and PC9 tumors in each treatment group. Images of isolated PC3 and PC9 tumors in each treatment group. PC3 tumors at day 24 and day 49 in each group were isolated (left panel) and PC9 tumors at day 25 and day 46 in each group were isolated (right panel). Their wet weight was shown in Fig.4C.

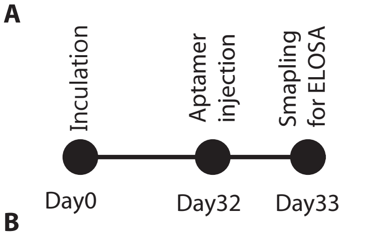

B

		n1	n2
Brain	saline	522.7	532.7
	Aptamer	514.5	534.9
Liver	saline	603.5	625.5
	Aptamer	687.5	632.4
Kidney (left)	saline	137.2	139.5
	Aptamer	138.5	141.2
Tumor	saline	326.2	269.7
	Aptamer	393.1	413.7

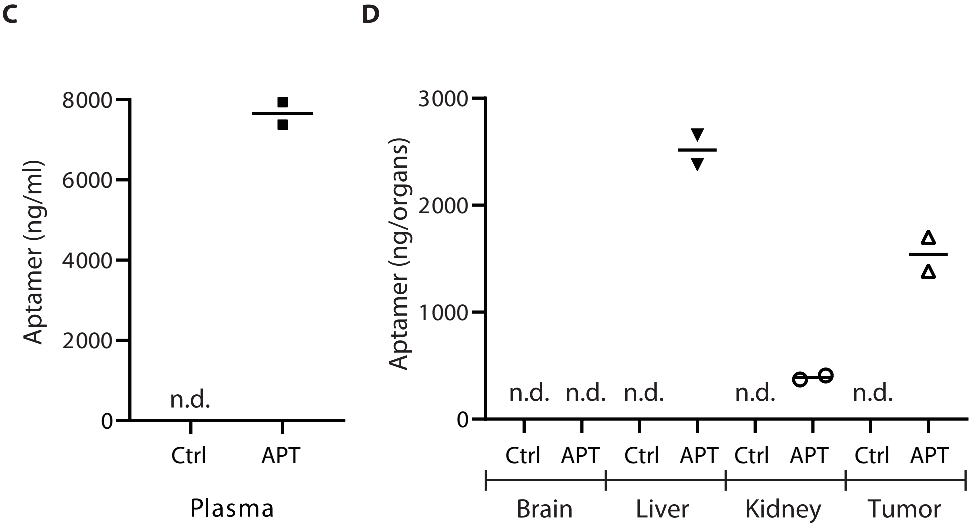

Fig. S13. Examination of deliver of aptamer to tumors by ELOSA assay. (A) Experimental schedule. PC9-luc cells were inoculated into left flank of NOD/SCID mice. At the day 32, aptamer APT-β1-OMe-P at a dose of 10 mg/kg or saline as a vehicle control (n=2/group) was subcutaneously injected to the mice bearing tumor. One day after the injection, concentrations of the aptamer in several organs, plasma and tumor were examined by ELOSA. (**B**) Wet weight of tissues. Wet weight of indicated tissues was measured. (**C**) Concentration of aptamer in plasma. Plasma concentrations of APT-β 1-OMe-P in mice treated with the aptamer or saline was shown (APT; ATP-β1-OMe-P injection [10 mg/kg], Ctrl; saline injection). (**D**) Concentration of aptamer in several tissues. Tissue concentrations of APT-β1-OMe-P in mice treated with the aptamer or saline were shown.

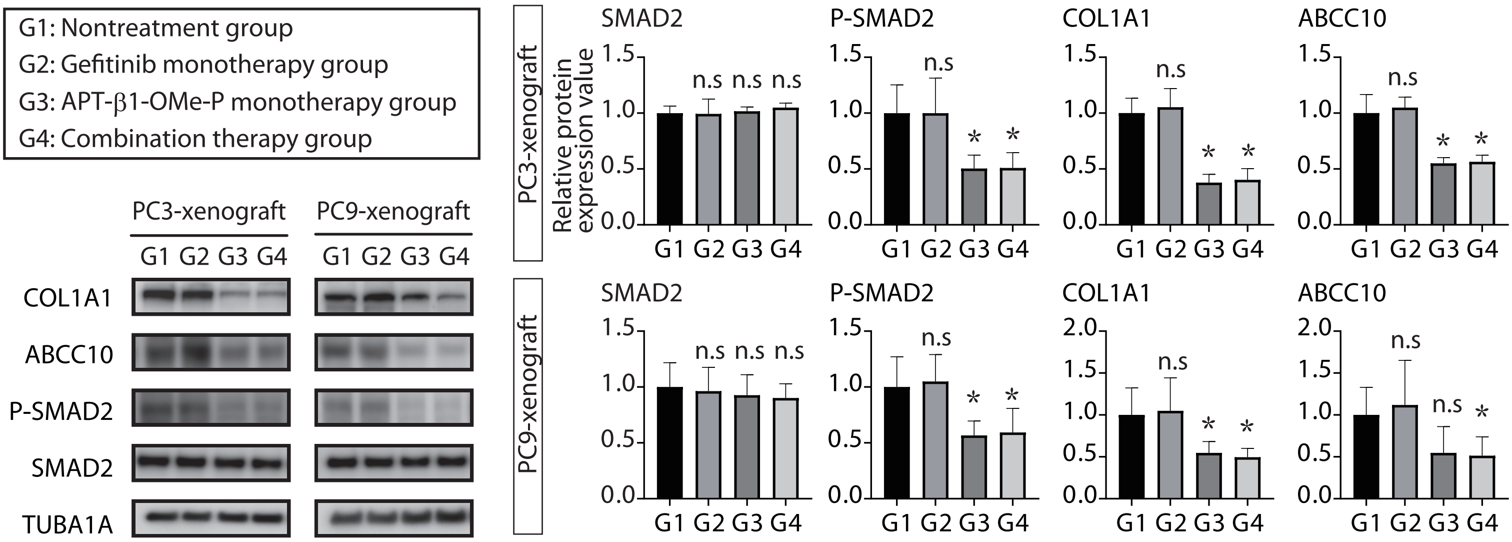

Fig S14. Altered protein expression levels of TGF-β1-induced genes in NSCLC xenograft tumors by APT-β 1-OMe-P. As in figure 3, the protein expression levels of TGF-β1-induced COL1A1 and ABCC10, and phosphorylation levels of SMAD2 in each treatment group of PC3 xenograft at day 24 and PC9 xenograft at day 25 were examined by western blot. The expression levels of those genes were normalized by the expression levels of TUBA1A1, and then the normalized values of each gene in the various treated cells were expressed as relative expression levels to those of control group in PC3 or PC9 xenograft without any treatments as one. Statistical differences among treatment groups were examined by one-way ANOVA and then by Tukey-Kramer test. Data represent the mean ± s.d. (n=5). * $P<0.05$ vs nontreatment gourp (G1) in each xenograft model, n.s. no significant difference vs nontreatment group (G1) in each xenograft model.

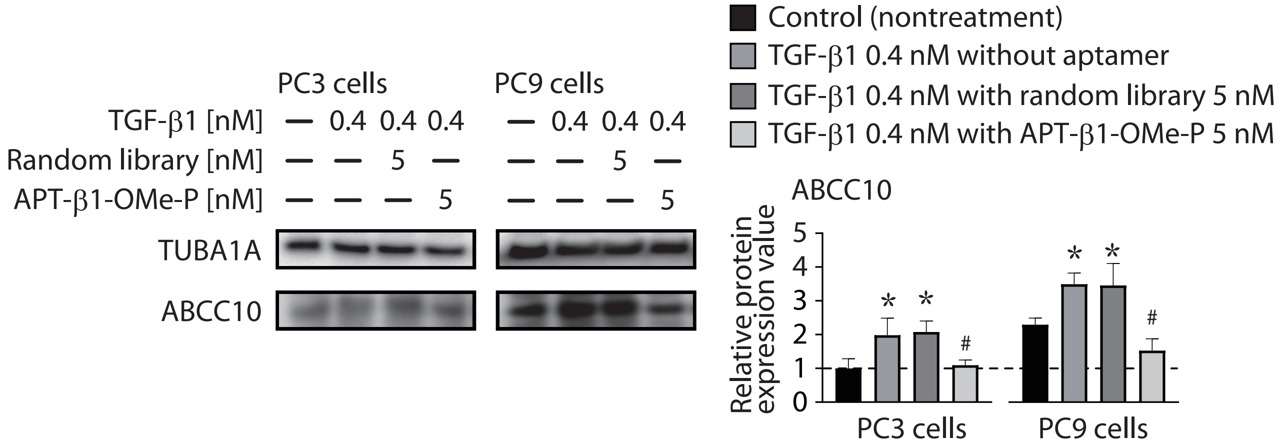

Fig. S15. Protein expression levels of ABCC10 in cultured NSCLCs. As in figure 3B, cultured NSCLCs, PC3 and PC9 cells, were treated with or without TGF-β1 (0.4 nM) in the presence or absence of APT-β1-OMe-P (5 nM) and random library (5 nM) as a negative control for 24 h, and the protein expression levels of ABCC10 gene were examined by western blotting. The protein expression levels of ABCC10 were normalized as in figure 3C. Statistical differences among indicated groups in each cell line were examined by one-way ANOVA and then by Tukey-Kramer test. Data represent the mean ± s.d. (n=3). * $P < 0.05$ vs nontreatment group in each cell type, # $P < 0.05$ vs treatment with random library and TGF-β1 in each cell type. Dashed line in the graph indicated the protein expression levels of ABCC10 in PC3 cells without any treatments.

Table S1. Resutls of ANOVA in Fig. 4C for PC3 xenograft

		Wet weight of tumors at day 24		Wet weight of tumors at day 49	
		F (DFn, DFd)	P value	F (DFn, DFd)	P value
Two-way ANOVA	Effect of APT-β1-OMe-P	F(1, 16)=8.618	0.0097	F(1, 16)=2.469	0.1357
	Effect of gefitinib	F(1, 16)=312.4	<0.0001	F(1, 16)=65.11	<0.0001
	Effect of gefitinib x APT-β1-OMe-P	F(1, 16)=0.0244	0.8778	F(1, 16)=0.00454	0.8339

Table S2. Resutls of ANOVA in Fig. 4C for PC9 xenograft

		Wet weight of tumors at day 25		Wet weight of tumors at day 46	
		F (DFn, DFd)	P value	F (DFn, DFd)	P value
Two-way ANOVA	Effect of APT-β1-OMe-P	F(1, 16)=0.02492	0.8765	F(1, 16)=2.843	0.1175
	Effect of gefitinib	F(1, 16)=103.8	<0.0001	F(1, 16)=59.48	<0.0001
	Effect of gefitinib x APT-β1-OMe-P	F(1, 16)=18.87	0.0005	F(1, 16)=0.6066	0.4512

Table S3 . Resutls of ANOVA in Fig. 4D for PC3 xenograft

		Ratio of luciferase activities of day 24 to day 36		Ratio of luciferase activities of day 36 to day 49	
		F (DFn, DFd)	P value	F (DFn, DFd)	P value
Two-way ANOVA	Effect of APT-β1-OMe-P	F(1, 16)=4.571	0.043	F(1, 16)=0.00044	0.9835
	Effect of gefitinib	F(1, 16)=11.78	0.0034	F(1, 16)=17.38	0.0007
	Effect of gefitinib x APT-β1-OMe-P	F(1, 16)=4.372	0.0528	F(1, 16)=3.542	0.0781

Table S4. Resutls of ANOVA in Fig. 4D for PC9 xenograft

		Ratio of luciferase activities of day 25 to day 39		Ratio of luciferase activities of day 39 to day 46	
		F (DFn, DFd)	P value	F (DFn, DFd)	P value
Two-way ANOVA	Effect of APT-β1-OMe-P	F(1, 12)=5.137	0.0427	F(1, 12)=0.3308	0.5758
	Effect of gefitinib	F(1, 12)=2.103	0.1727	F(1, 12)=0.0094	0.9241
	Effect of gefitinib x APT-β1-OMe-P	F(1, 12)=4.650	0.052	F(1, 12)=4.372	0.0597
